# Calcium Signaling in ß-cell Physiology and Pathology: A Revisit

**DOI:** 10.3390/ijms20246110

**Published:** 2019-12-04

**Authors:** Christiane Klec, Gabriela Ziomek, Martin Pichler, Roland Malli, Wolfgang F. Graier

**Affiliations:** 1 Division of Oncology, Department of Internal Medicine, Medical University of Graz, 8036 Graz, Austria; christiane.klec@medunigraz.at (C.K.); martin.pichler@medunigraz.at (M.P.); 2 Research Unit Non-Coding RNAs and Genome Editing in Cancer, Medical University of Graz, 8010 Graz, Austria; 3 Molecular Biology and Biochemistry, Gottfried Schatz Research Center for Cell Signaling, Metabolism and Aging, Medical University of Graz, Neue Stiftingtalstraße 6/6, 8010 Graz, Austria; gabiziomek@yahoo.com (G.Z.); roland.malli@medunigraz.at (R.M.); 4 BioTechMed Graz, 8010 Graz, Austria

**Keywords:** β-cells, Ca^2+^ signaling, insulin secretion, diabetes, mitochondria

## Abstract

Pancreatic beta (β) cell dysfunction results in compromised insulin release and, thus, failed regulation of blood glucose levels. This forms the backbone of the development of diabetes mellitus (DM), a disease that affects a significant portion of the global adult population. Physiological calcium (Ca^2+^) signaling has been found to be vital for the proper insulin-releasing function of β-cells. Calcium dysregulation events can have a dramatic effect on the proper functioning of the pancreatic β-cells. The current review discusses the role of calcium signaling in health and disease in pancreatic β-cells and provides an in-depth look into the potential role of alterations in β-cell Ca^2+^ homeostasis and signaling in the development of diabetes and highlights recent work that introduced the current theories on the connection between calcium and the onset of diabetes.

## 1. Introduction

Under physiological conditions, blood glucose levels only transiently increase following intake of food [1]. The observed rise in blood glucose is controlled through the body’s employment of insulin, a hormone generated by the beta (β) cells of the pancreatic islets of Langerhans. Insulin’s primary function is to regulate the body’s metabolism of carbohydrates, protein, and fats. It does so by fostering the absorption of carbohydrates (primarily glucose) out of the blood into skeletal muscle, adipose tissue and the liver [2].

The islets of Langerhans are structures found throughout the exocrine pancreas, and together they form the endocrine part of this gland. They contain primarily β, alpha (α), gamma (γ), and delta (δ) cells. Each of these cell types secretes a separate hormonal product (insulin, glucagon, pancreatic polypeptide, and somatostatin) and they are evident in different proportions across species [3]. These cell types are all able to communicate with each other through paracrine signals, integral to the intricate web of signaling and maintenance of β-cell health [4].

Key to the role of insulin in maintaining homeostatic blood glucose levels is the sensitivity of β-cells to the glucose itself [5]. For example, when blood glucose increases following food intake, β-cells sense this change in concentration and subsequently secrete insulin into the blood [6]. On the other hand, when blood glucose levels are low, such as following a prolonged fasting period, the release of insulin from β-cells is inhibited [7]. Importantly, insulin’s effect on glucose metabolism directly counteracts those of multiple hormones, such as glucagon [8]. The interplay between the activity of hormones like insulin and glucagon, therefore, regulates overall glucose homeostasis in the body.

Pathophysiological conditions arise with insulin dysregulation; declines in either insulin’s release or its actual production by β-cells characterizes the main features of diabetes mellitus (DM) development. Diabetes is a significant health problem: according to the International Diabetes Federation close to 10% of the worldwide adult population is affected by this disease [9]. Type 1 diabetes (T1DM) is the result of decreased insulin production by the pancreas due to destroyed β-cells. This typically occurs as a result of an autoimmune reaction in the body and results in insulin not being able to be synthesized or subsequently secreted into the blood [10]. Type 2 (T2DM), in comparison, is characterized by less β-cell destruction than its type 1 counterpart, as cells are not destroyed through an autoimmune response [11]. This form of diabetes is observed when remaining functional β-cells are unable to produce insulin in sufficient amounts to compensate for the body’s insulin resistance [12].

Calcium (Ca^2+^) has a significant role to play in physiological insulin release from islet β-cells. Glucose, acting as the main stimulus of these cells, actually serves to control Ca^2+^ concentrations across many compartments of the cell, including the endoplasmic reticulum (ER), mitochondria, nucleus, and cytosol, among others. Ca^2+^ dysregulation in β-cells also has been observed in disease states, including type 2 diabetes. The present review discusses previously known, as well as recently emerged aspects of Ca^2^ dynamics in pancreatic β-cells, and how this new information may relate to the pathophysiological development of T2DM.

## 2. The Role of Ca^2+^ Signaling in Pancreatic β-cells: The Basics

**Figure 1:**
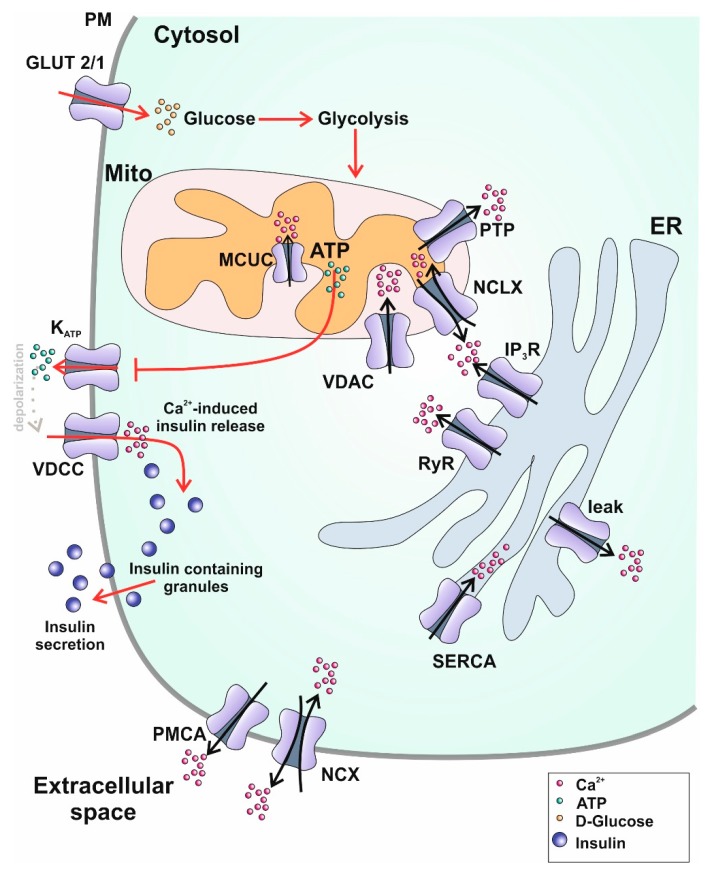
Schematic representation of glucose stimulated insulin secretion (GSIS) and subcellular Ca^2+^ dynamics in pancreatic β-cells. Ca^2+^ transporters within a pancreatic β-cell responsible for balancing Ca^2+^ homeostasis are the following: in the plasma membrane: PMCA: plasma membrane Ca^2+^-ATPase, NCX: Na^+^/Ca^2+^ exchanger; within the endoplasmic reticulum: SERCA: sarco/endoplasmic reticulum Ca^2+^-ATPase, RyR: ryanodine receptor, IP_3_R: inositol 1,4,5-trisphsphate receptor, VDAC: voltage dependent anion-selective channel; within mitochondria: NCLX: mitochondrial Na^+^/Ca^2+^ exchanger, PTP: permeability transition pore, MCUC: mitochondrial Ca^2+^ uniporter complex. Red arrows depict the process of glucose-stimulated insulin secretion. After uptake of glucose via GLUT2/1 mitochondrial ATP production is boosted leading to a closing of plasma membrane located ATP-sensitive K^+^ channels (K_ATP_). The resulting shift in membrane potential activates PM voltage-dependent Ca^2+^ channels (VDCC) stimulating Ca^2+^-induced Ca^2+^ release which ultimately leads to exocytosis of insulin-containing granules.

The movement of Ca^2+^ is vital to the function of any cell type but has a special role to play in pancreatic β-cells due to its importance in the process of insulin release. Insulin production and subsequent release from these cells is controlled by multiple players, including glucose, neurotransmitters, peptide hormones, and other compounds [13,14]. Briefly, the elevation in blood glucose levels that follows food intake is sensed by these cells, which subsequently take glucose up from the blood and metabolize it to more fuel for the mitochondria to shunt towards ATP production, increased levels of which result in the inhibition of the cell’s K_ATP_ channels. This ultimately leads to depolarization at the plasma membrane (PM), an electrical change that functions to activate L-type Ca^2+^ channels, which allows an influx of Ca^2+^ into the cell. Finally, this wave of Ca^2+^ triggers the release of secretory granules containing insulin, to be released from the cell by exocytosis (Figure 1).

Previous work has also put forth that glucose may directly affect the activity of voltage-dependent Ca^2+^ and K^+^ channels [15]. Recent investigation has also shown that cellular insulin secretion stimulated by glucose is tied to mitochondrial Ca^2+^ dynamics. Namely, the proper functioning of mitochondrial Ca^2+^ uptake 1 (MICU1) and mitochondrial calcium uniporter (MCU) proteins is necessary for the physiological function of β-cells [16].

The well-established insulin release-triggering pathway outlined above is not the only mechanism wherein glucose stimulates insulin release. Another, as yet not completely understood pathway is also evident, though it depends on the K_ATP_-dependent mechanism due to the need for high cytosolic Ca^2+^ levels. Therefore, it is poignant to consider the fact that any of β-cells’ channels that may affect membrane potential could also have an effect on Ca^2+^ movement into the cytosol and thus insulin secretion.

Importantly, β-cells have a wide array of channels involved in Ca^2+^ influx across their plasma membranes. The placement of these excitable cells’ voltage-dependent channels has been observed to be erratically distributed across the PM, allowing for micro-domains containing high levels of Ca^2+^ to form [17]. In certain species, the electrical activity of the β-cells contained within an islet as a whole is synchronized due to coupling through the presence of gap junctions [18]. This leads to synchronized Ca^2+^ oscillations between the cells as well, which subsequently induce insulin secretion waves observable at the level of a single islet. This does not apply fully to human islets, because while there is some coupling apparent between adjacent β-cells, there is a more heterogeneous spread of the many cell types present in each islet, thus affecting the ability of β-cells separated by other cell types to behave synchronously [18].

The extrusion of Ca^2+^ from β-cells does not differ significantly from the majority of other cell types. Ca^2+^ in the cytosol is removed through a combination of Na^+^/Ca^2+^ exchanger(s) (NCX) and plasma membrane Ca^2+^–ATPase(s) (PMCA) activity [19,20,21,22]. As each of these transporters have complementary characteristics (PMCA has a high affinity for Ca^2+^ and a low extrusion capacity, while NCX displays exactly the opposite traits [23]), each exhibits different levels of activity under cellular conditions. Namely, NCX acts as the main Ca^2+^ extrusion pathway at high cytosolic Ca^2+^ concentrations, while PMCA takes over most extrusion activity when low levels are observed [24].

ER Ca^2+^ homeostasis is vital to proper function in most cell types [25]. In the case of β-cells, Ca^2+^ is taken up by the ER through SERCA activity, specifically isoforms SERCA2b and SERCA3, which are expressed ubiquitously and only in β-cells found within pancreatic islets, respectively [26]. Insofar as Ca^2+^ release from the ER goes, this organelle is known to exhibit considerable Ca^2+^ leakage into the cytosol, which must ultimately be corrected for by SERCA pump activity [27]. A likely mechanism for the ER Ca^2+^ leak in β-cells was recently outlined to involve presenilin-1 (PS 1) [28].

As in various other cell types, ER Ca^2+^ release in β-cells is mediated by inositol 1,4,5-triphosphate (IP_3_R) and ryanodine receptors (RyR) [29]. The latter can be activated by (local) Ca^2+^ elevations representing the so-called “calcium-induced calcium release” (CICR) processes [30,31]. In regard to the Ca^2+^ release from the ER in pancreatic β-cells, it is unclear whether they exhibit CICR as triggered by RyRs on the ER membrane. These cells show expression of all three of IP_3_R isoforms, but their expression and the overall function of RyR is reported as being underwhelming [32,33]. It has been discussed that RyR expression in β-cells is much lower than in most other tissues. Nevertheless, it is possible that lower expression of RyR may be sufficient to allow β-cells’ to accomplish CICR, because of their large-conductance capacity [34].

Another very important aspect of pancreatic β-cell Ca^2+^ signaling is the great role of stromal interaction molecules 1 (STIM1/2) and its pairing with Orai1/2/3. Generally speaking, Orais are ion channels that are selective for Ca^2+^, and are activated following this ion’s depletion from intracellular Ca^2+^ stores [35,36]. The depletion of ER Ca^2+^ is first sensed by the ER STIM1 protein, which is then induced to oligomerize at ER/plasma membrane junctions. STIM1 subsequently activates the Orai1 Ca^2+^-release-activated channel (CRAC) through direct protein interaction, thus inducing store-operated Ca^2+^ entry (SOCE) [37]. STIM has been observed to have two highly homologous isoforms, STIM1 and STIM2. Despite their degree of similarity, the two isoforms have differing functions. Namely, STIM1 serves as the main activator of SOCE channels, while STIM2 serves as a feedback regulator maintaining ER and cytosolic Ca^2+^ concentrations within a narrow range [38,39]. The Orai protein also exists in various forms: Orai1, Orai2, and Orai3. Orai2 and 3 are not as thoroughly characterized as Orai1, but they have been shown to have similar roles in modifying SOCE across different cell types [40,41,42].

Since the discovery of its mechanism in the early 2000s, the STIM/Orai Ca^2+^-signaling pair has been found to play a distinct role in pancreatic β-cell insulin secretion. It has been demonstrated in rat β-cells that a complex is formed by Orai1, STIM1, and TRPC1 proteins in response to ER Ca^2+^ depletion [43]. Blocking the activity of Orai1 or TRPC1 was shown to impair GSIS in these cells [44], indicating that Orai1 and TRCP1 are vital to the formation of store-operated Ca^2+^ channels (SOCs), which, combined with their activation by STIM1, are necessary for the physiological response of cells to acetylcholine (Ach) in insulin secretion. Furthermore, the group postulated that the effective activation of these SOCs may be reduced in T2DM, thus confirming the importance of Orai1 to the pancreatic cell’s optimal functionality [44]. Notably, the importance of basal mitochondrial Ca^2+^ entry, possibly via a TRPC-mediated mechanism has been postulated to be fundamental for the responsiveness to increased glucose [28].

Beyond the basic mechanisms involved in β-cell Ca^2+^ signaling, the bigger picture must of course also be considered. What kind of signals are these channels and pumps propagating? What effects can they exert on the cell? Ca^2+^ sparks, the creation of Ca^2+^ microdomains, the potential effect of Ca^2+^ on processes ranging from channel activity to gene expression are but a few of the many ways that this ion exerts its effect on β-cell function. Oscillations are also observable in β-cells, as well as almost universally across cell types, including cells performing substantially varied functions, such as fibroblasts and astrocytes [45,46,47]. Importantly, glucose-stimulated insulin release has been associated with transient changes to these cells’ cytosolic Ca^2+^ concentration. This increase in Ca^2+^ levels, and the subsequently observed oscillations are induced by cellular glucose metabolism [48]. It has been determined that in primary mouse islets, for example, the addition of glucose to cells can induce the generation of IP_3_. Every time that cytoplasmic Ca^2+^ increased, so did IP_3_, indicating clearly that these processes are related [45].

Cellular Ca^2+^ oscillations have also been linked to effects on gene expression. For example, Dolmetsch and colleagues [49], demonstrated that cytoplasmic Ca^2+^ oscillations effectively reduced the Ca^2+^ threshold required for the activation of a specific set of transcription factors. Whether this holds true in β-cells is as yet unclear, though other breakthroughs in the role of Ca^2+^ in β-cell gene expression are evident in the literature. It was discussed that the signals resulting from glucose flux into the cells are transmitted through the insulin enhancer, activation of which could result from direct protein modification along the lines of phosphorylation or similar processes, or through changes in local co-factor concentrations through Ca^2+^ signals [50]. Others have shown that extended exposure of β-cells to glibenclamide, an ATP-sensitive K^+^-channel inhibitor, which depolarizes the β-cells and thereby stimulates the cells to secrete insulin (often enlisted as a treatment tool for diabetes) [51], causes a prolonged increase in the cells’ basal insulin production, and showed that this was a Ca^2+^-dependent effect [52]. Ultimately, it was determined that extended exposure of the cells to this insulin secretagogue activated protein translation through Ca^2+^-regulated signaling pathways mTOR, MEK, and PKA [52].

The important role of Ca^2+^ in β-cells’ general function, indicates that dysregulation of this cation likely contributes strongly to the development of pathophysiological conditions.

## 3. The Role of Ca^2+^ Signaling in Pancreatic β-Cells: A Closer Look

### 3.1. Ca^2+^ in β-Cell Proliferation

β-cells can adapt to increased metabolic demands to ensure balanced euglycemia by boosting their proliferation rates. Several studies demonstrate that besides elevated glucose levels also Ca^2+^ signaling is a prerequisite for inducing augmented β-cell replication [53,54,55]. Porat et al. could show that Ca^2+^ influx is essentially contributing to β-cell replication since blocking membrane depolarization with diazoxide drastically reduced β-cell proliferation rates [56]. Moreover, treating rat β-cells with the L-VGCC agonist BayB8644, thus, augmenting intracellular Ca^2+^ concentrations, stimulates β-cell proliferation [57,58].

Intracellular Ca^2+^ elevations promote β-cell proliferation amongst others by Ca^2+^/calmodulin dependent kinase 4 (CaMK4) and calcineurin/nuclear factor of activated T-cells (NFAT)-dependent mechanisms. In β-cells CaMK4 is activated by elevated glucose levels and increased intracellular Ca^2+^ levels [59]. Inhibition of CaMK activity or alterations in its expression influences glucose-mediated increase in β-cell proliferation. Liu et al. [60] demonstrated that CaMK inhibition or expression of a dominant-negative CaMK result in abolished β-cell proliferation rates whereas overexpression of a constitutive active version of CaMK leads to the opposite effect. The calcineurin/NFAT axis controls islet responses by promoting the expression of cell cycle regulators and increased β-cell proliferation rates and mass. NFAT family members control cell cycle regulators on the transcriptional level in β-cell which play a role in β-cell proliferation. Blocking NFAT inhibitory kinases such as DYRK1A and GSK3β supports and boosts β-cell proliferation [57,58]. Overexpression of NFATC2 in mice leads to 2-fold increased proliferation rates [61], an effect that has also been detected when the expression of NFATC1, NFATC3, and NFATC4 is increased [62]. β-cell-specific deletions of the Ca^2+^-activated calcineurin phosphatase regulatory subunit, calcineurin b1 (Cnb1), in mice results in the development of age-dependent diabetes characterized by reduced β-cell proliferation and mass, reduced pancreatic insulin content and hypoinsulinaemia [61]. Recent human data show that the ability of β-cells to replicate is highest in infancy, with their ability to regeneration declining substantially soon after this period of life [63]. This age-related decrease in proliferation of β-cells has also been corroborated in rodent models [64,65]. This, of course, begs the question: what is the expected life span of human β-cells? In vitro work has demonstrated that in culture, these cells are capable of releasing insulin for more than 9 months at a time [66].

### 3.2. Ca^2+^ in β-cell Survival

Ca^2+^ signals play a vital role in the induction of cellular apoptosis, a trigger event that occurs in response to a wide range of cellular conditions or particular intracellular agents. On the other hand, it is also possible for Ca^2+^ signals to function as a cell’s saving grace by ensuring cell survival [67]. Isolated mouse islet β-cells and MIN6 insulinoma cells exhibit 3-fold reduced apoptosis rates when cultured with high glucose medium (15 mM or 25 mM) compared to low-glucose medium (2 mM or 5 mM, respectively). The high glucose concentration increases survival-required depolarization and subsequent Ca^2+^ influx. These pro-survival effects get lost when depolarization is inhibited with diazoxide or Ca^2+^ influx with nifedipine [68]. As for β-cell proliferation, also β-cell survival is mediated by calcineurin and CaMK-pathways. Calcineurin inhibition has toxic effects on β-cells and promotes β-cell failure after transplantation by blunting β-cell replication [69] and apoptosis induction [70]. Simultaneous calcineurin inhibition and treatment with the mTOR inhibitor rapamycin impair β-cell regeneration during recovery from diphtheria toxin-induced β-cell death [71]. Conversely, calcineurin inhibition has been shown to be beneficial after treatment with cytokines [72] and corticosteroids [73]. These conflicting data prove that effected down-stream pathways are diverse, but the crucial involvement of Ca^2+^ in β-cell survival stays unchallenged. Furthermore, the ER Ca^2+^ homeostasis has been shown to impact β-cell survival. Several groups provide evidence that ER Ca^2+^ depletion results in ER stress and β-cell apoptosis [74,75,76]. This ER Ca^2+^ depletion occurs due to inhibition of the sarco/endoplasmic reticulum Ca^2+^ ATPase (SERCA) and the resultant reduced ER Ca^2+^ uptake in the situation when low glucose exposure is paralleled with low intracellular Ca^2+^ levels [77].

### 3.3. Ca^2+^ Oscillations for Proper β-Cell Function

The cycling variations of circulating insulin are reflected by the pulsatile release of the hormone from the pancreas [78].These insulin pulses occur over 4–13 min [79,80] whereas the rise of plasma glucose levels is preceding the rise in plasma insulin levels in average two minutes [81]. Loss of these regular insulin oscillations is an early phenomenon in the development of insulin [82] and non-insulin dependent [83] diabetes mellitus. Glucose stimulates an increase in cytosolic Ca^2+^ concentration in β-cells due to cellular depolarization mediated by closure of the ATP-sensitive K^+^-channels and subsequent opening of VDCC. This rise in [Ca^2+^]_i_ is not a steady-state increase of the ion, but rather consists of periodic Ca^2+^ oscillations [84,85]. These Ca^2+^ waves are synchronized with β-cell bursting electrical activity [86]. The basis for a proper insulin secretion upon increased glucose levels is a tightly controlled inter-islet regulation, which are coupled by gap junctions. Islet β-cells are comprised of functionally heterogeneous cell populations, whereas a special subpopulation termed “hubs” is crucial for mediating the islets response to high glucose levels. Inhibition of these hubs abolished the coordinated and tightly regulated Ca^2+^ oscillations necessary for driving insulin secretion [87]. Controlled by these hubs, the islets behave as functional syncytium in response to glucose and therefore homogenizing the actual heterogeneous cell population [88]. Diverse oscillation patterns are existing: the fast, the slow (approx. 5 min) and the mixed oscillations [48]. Slow activated Ca^2+^-sensitive K^+^-channels and cyclic cytoplasmic Na^+^ changes are responsible for the fast and the slow oscillations, respectively. The ATP/ADP ratio and the ER Ca^2+^ levels are supposed to serve as pacemakers for fast bursts and cytosolic Ca^2+^ oscillations [89]. The total secretory activity is determined by the number of β-cells recruited into the active phases [90]. Several physiologic advantages of the glucose-induced Ca^2+^ oscillations within β-cells should be mentioned. As these Ca^2+^ oscillations are the basis for the fluctuating plasma insulin signals, a down-regulation of peripheral receptors is prevented. This is substantiated by the finding that the recycling time for these receptors is shorter than the periods for plasma insulin pulses [91]. Furthermore, the intracellular Ca^2+^ pulses prevent the desensitization of the secretory machinery of the β-cells. Jones et al. [92] could show that prolonged exposure to high Ca^2+^ concentrations in permeabilized β-cells makes the machinery refractory to Ca^2+^ signals. A steady-state rise of cytosolic Ca^2+^ concentrations in stimulated β-cells could be responsible for the loss of the pulsatile insulin signals, a process occurring at the onset of diabetes [82,83]. Sustained Ca^2+^ rise may activate autolytic processes by stimulating phospholipases, proteases and endonucleases [93] deteriorating primary lesions of a diabetic β-cell [90].

### 3.4. Ca^2+^ in Biphasic Insulin Secretion

Nowadays, it is well established that insulin secretion follows a biphasic pattern which was clearly described for the first time in perfused rat pancreas in 1968 [94] and one year later in humans [95]. The early peak is starting 3–5 min after glucose stimulation lasting for approximately ten minutes. This rapid insulin peak is followed by a sustained period of slowly increasing insulin levels lasting as long as the glucose level is elevated [96]. In patients diagnosed with T2DM this initial peak is absent and the second phase reduced or delayed [97,98,99,100]. The early phase is pivotal for human physiology due to several processes initiated by this first rise in insulin such as suppression of hepatic glucose production [101,102], suppression of lipolysis [102] and preparation of target cells i.e., liver, muscle, and adipocytes—to the action of insulin [103]. The reduction of the first-phase of insulin secretion occurs early in disease. The involvement of Ca^2+^ in the process of (biphasic) insulin secretion is unchallenged but early studies on its contribution showed a great extent of controversy: Wollheim et al. [104] stated that the first phase insulin response is independent of glucose-stimulated uptake of extracellular Ca^2+^ but crucially depends on intracellular Ca^2+^ handling. Contrary findings were presented by Henquin et al. [105] showing that withdrawal of extracellular Ca^2+^ or inhibition of Ca^2+^ influx abolishes glucose-stimulated insulin secretion postulating that extracellular Ca^2+^ influx plays an essential role for insulin secretion. The current model of Ca^2+^ signaling in insulin secretion attributes important functions to both theories. Ca^2+^ influx through voltage-dependent Ca^2+^ channels is a prerequisite for proper insulin exocytosis [106] but also intracellular Ca^2+^ flux is necessary for physiological insulin secretion. The latter recently has been outlined by our group as we demonstrated a pivotal engagement of a β-cell-specific permanent presenilin-1-mediated ER-Ca^2+^ leak in the initiation of first-phase insulin secretion. Inhibition of presenilin-1 activity or reduction of its expression or in other words a reduction of the ER Ca^2+^ leak leads to abrogation of the first phase of insulin release [107].

## 4. Ca^2+^ on Subcellular Level

Cytoplasmic Ca^2+^ homeostasis in β-cells is tightly regulated by a complex interplay between Ca^2+^ uptake and extrusion across the plasma membrane as well as uptake and release into/from internal Ca^2+^ stores. Since alterations in this equilibrium result in drastic changes of Ca^2+^ signaling in β-cells ultimately affecting insulin secretion, it is of crucial importance to keep cellular Ca^2+^ fluxes balanced. In the next paragraph, the most important contributors to β-cell Ca^2+^ homeostasis are discussed.

### 4.1. Plasma Membrane

As described above, Ca^2+^ transporters regulating Ca^2+^ extrusion from the cell—such as NCX and PMCA—located within the plasma membrane, function as essential players in maintaining a balanced Ca^2+^ homeostasis in β-cells. Notably, the activity of the forward-mode (i.e., 1Ca^2+^ out/3Na^+^ in) of the NCX in the pancreatic β-beta-cell is sensitive to long-chain acyl-coenzyme and alterations in intracellular acyl-CoA levels may cause negatively control Ca^2+^-mediated exocytosis and insulin secretion [108]. Moreover, the forward mode of the NCX1 splice variants of that is expressed in the pancreatic β-cells (NCX1.3 and NCX1.7) exhibit an unusually high sensitivity to KB-R7943 [109] that rather selectively inhibits the reversed mode of the NCX (i.e., 1Ca^2+^ in/3Na^+^ out) in most other cell types. Consequently, the pharmacological inhibition of the NCX1 with KB-R7943 results in a considerable augmentation in glucose-stimulated increases in cytosolic Ca^2+^ concentrations and insulin secretion in mouse and human β-cell islets, while KB-R7943 was without effect under non-stimulated conditions. [109]. Heterozygous inactivation of PMCA isoform 2 (PMCA2) in mice also leads to cytosolic Ca^2+^ accumulation and to a 1.5-fold increase in glucose-induced insulin release. Furthermore, PMCA2 inhibition results in increased β-cell mass, proliferation, and viability [110].

### 4.2. Mitochondria

Cellular energy demand and mitochondrial activation are tightly linked especially in states of energy-consuming processes. Increased energy demand especially observed during stimulation of a cell—comes along with enhanced ATP hydrolysis which must be compensated for by accelerated ATP synthesis. Examples for such an increased energy demand where mitochondria are involved in balancing the energetic homeostasis are increased workload of the heart [111], activation of specific brain areas [112] and insulin production in pancreatic β-cells after glucose stimulus. Upon glucose stimulation, β-cell mitochondria augment oxidative metabolism [113], respiration [114,115] and mitochondrial ATP synthesis rate [116] to match the increased energy need. Hormones and extracellular messengers that stimulate ATP-requiring processes through rises of cytosolic Ca^2+^ concentrations regulate oxidative metabolism whereas the Ca^2+^ concentration needed for proper stimulation of the downstream processes ranges from 0.1–10 µM. The citric acid cycle (i.e., the Krebs cycle) is the major energy-producing metabolic pathway in mitochondria and generally in cells, providing enormous amounts of reduction-equivalents which subsequently are oxidized in the electron transport chain to yield ATP. Ca^2+^ was shown to regulate three key dehydrogenases of the citric acid cycle i.e., NAD^+^-isocitrate dehydrogenase (NAD-ICDH), 2-oxoglutarate dehydrogenase (OGDH) and pyruvate dehydrogenase (PDH) [117,118,119]. Notably, because of its stimulatory effect on the dehydrogenases of the citric acid cycle [120], mitochondrial Ca^2+^ increase is known to serve as a key trigger for insulin release in β-cells [121]. This offers some contradiction to the current concept of at exactly what point in the process of GSIS mitochondrial Ca^2+^ is involved. In fact, the current concept suggests that mitochondrial Ca^2+^ increase occurs upon Ca^2+^ entry due to the opening of the L-type Ca^2+^ channels, thus, already downstream from mitochondrial activation by glycolysis-derived pyruvate and ATP production. However, increases in mitochondrial Ca^2+^ are thought to be essential for stimulation of Ca^2+^-dependent dehydrogenases of the citric acid cycle in order to establish suitable enzyme activity to handle the substrate overflow upon elevated blood D-glucose [122]. In particular, the responsiveness and proper D-glucose sensing of β-cells to elevated blood D-glucose is determined by the cells’ pace of producing ATP when exposed to elevated glucose [123]. Data of our group show strongly enhanced basal respiratory activity and elevated mitochondrial ATP levels in mitochondria of clonal β-cell lines in comparison to non-β-cell lines pointing to such pre-stimulation of mitochondria in β-cells [28]. These data are in line with the new perspective of GSIS that involves a continuous priming of β-cells based on a weak stimulation of β-cell mitochondria to ensure proper responsiveness to elevated blood D-glucose sensing [124].

### 4.3. Endoplasmic Reticulum

The endoplasmic reticulum as preserver of Ca^2+^ homeostasis in β-cells plays an important role in β-cell physiology and insulin secretion. ER stress caused by ER Ca^2+^ depletion, has been shown to contribute to β-cell dysfunction and the development of type 2 diabetes [125]. The Ca^2+^-sensor protein sorcin, which is a 22 kDa protein of the penta EF-hand family, is involved in maintaining ER Ca^2+^ stores by inhibiting RyR-activity and playing a role in the termination of CICR [126]. Sorcin is strongly expressed in primary mouse islets [127]. Knock-down in MIN6 insulinoma β-cells reduces ER Ca^2+^ stores and inhibits GSIS [128]. Elevated Ca^2+^ levels lead to a conformational change of the sorcin protein, a subsequent translocation from the cytoplasm to membranes (amongst others to the ER) where it interacts with target proteins including RYR [126], thereby, inhibiting RYR [126,129] and activating SERCA pumps [130].

Binding of insulin to the insulin receptor at the cell surface leads to autophosphorylation of tyrosyl residues of the IR-β-subunit and the phosphorylation of insulin receptor substrates 1 and 2 (IRS1 and 2). The subsequent cascade initiates the translocation of the insulin-responsive glucose transporter GLUT4 in muscle cells and adipocytes [131]. In β-cells IRS-1 is able to translocate to several intracellular locations dependent on upstream signals, which is also true for intracellular membranes, in particular, the ER [132]. The β-cell-selective insulin receptor (IR) knockout and IRS-1 knockout result in reduced glucose-induced insulin secretion while overexpression of IR and IRS-1 result in augmented insulin secretion and increased cytosolic Ca^2+^ levels due to inhibition of the ER-located SERCA. In β-cells the SERCA2 and SERCA3 are expressed [26]. Loss of SERCA activity and reduced SERCA3b expression in β-cells are associated with diabetes development in *db/db* mice [20]. SERCA3 gene mutations (Gln108→His, Val648→Met, Arg674→Cys and Ile753→Leu) in type 2 diabetes patients provide a link to a genetic susceptibility to T2DM development [133].

The above-mentioned ER Ca^2+^ depletion and subsequent ER Ca^2+^ stress can be explained—at least partially—by the existence of ER Ca^2+^ leak channels. Cassel and Ducreux [27] could show that translocon-mediated ER Ca^2+^ leak in murine MIN6 insulinoma β-cells and human islets is influencing lipotoxicity. Furthermore, translocon inhibition resulted in reduced ER stress and a restoration of insulin secretion [27]. Two recent studies of our group demonstrated that a presenilin-1-mediated ER Ca^2+^ leak crucially contributes to β-cell physiology and insulin secretion. The presenilin-1-mediated ER Ca^2+^ leak is directly sequestered by mitochondria, leading to increased basal matrix Ca^2+^ levels that yield enhanced resting activity of mitochondria in the pancreatic β-cells due to pre-stimulation the Ca^2+^-dependent dehydrogenases of the citric acid cycle. Upon elevation of glucose, glucose is metabolized and the pre-activated citric cycle in the mitochondria efficiently converts glucose metabolism to activation of the respiratory chain (OXPHOS) and, subsequently, fast ATP production, thus, ensuring a fast, initial insulin secretion within 10 min of exposure to elevated glucose [28,107].

### 4.4. The Golgi-Apparatus

Another intracellular Ca^2+^ storage important for a balanced Ca^2+^ homeostasis in mammalian cells and also in β-cells is the golgi apparatus. IP_3_ receptors are expressed at the surface of the golgi apparatus, mediating Ca^2+^ release from these IP_3_-sensitive pools [134]. Early measurements of intracellular Ca^2+^ demonstrate that upon cellular stimulation with IP_3_-generating agonists such as histamine, the golgi Ca^2+^ concentration rapidly decreases, presenting the golgi apparatus as IP_3_-sensitive Ca^2+^ pool [135]. However, in cell types that exhibit a high expression of RyR (such as cardiac myocytes), the Ca^2+^ extrusion of the golgi apparatus is mediated by these receptors [136]. The ATP-sensitive Ca^2+^ pump responsible for fueling the golgi apparatus with Ca^2+^ from the cytoplasm is the secretory pathway Ca^2+^-ATPase Ca^2+^ pump (SPCA1) [134]. Two main isoforms of this Ca^2+^ pump exist i.e., SPCA1 and SPCA2, whereas they show a tissue-specific expression. In mammals, SPCA1 is expressed in all tissues [137] whereas SPCA2 is expressed only in a limited set of tissues [138]. SPCA1 has been identified as being the main regulator of golgi Ca^2+^ homeostasis [139], which is also true for pancreatic β-cells [140]. Bone et al. [140] demonstrated a crucial role of SPCA1 in β-cell physiology. On the one hand, SPCA1 expression is reduced in patients suffering from T1DM and T2DM and on the other hand SPCA1 knock-out β-cells show increased rates of apoptosis, augmented cytosolic Ca^2+^ levels and significantly reduced GSIS (bone), highlighting the importance of the Ca^2+^ homeostatic function of the golgi apparatus.

## 5. Ca^2+^ in the Development of T2DM

As described in parts three and four of this review, Ca^2+^ is a crucial factor for β-cell survival, proliferation and function as well as for a proper insulin secretion on the one hand and is tightly regulated among diverse intracellular compartments within β-cells on the other hand. Therefore, an association with the development and progression of diabetes is obvious. Furthermore, deregulated Ca^2+^ signaling has been associated with the development of one of the key characteristics of T2DM i.e., insulin resistance [141,142,143]. Deregulated Ca^2+^ homeostasis has been implicated in a vast range of disease conditions. The case is not different when considering T2DM. In fact, it seems likely that there can be multiple degrees of separation between an original Ca^2+^ dysregulation and the eventual development of T2DM. In this section some issues of how Ca^2+^ deregulation can contribute to diabetes pathophysiology by highlighting some Ca^2+^-associated aspects on the cellular level but also in the human body.

Problems with pancreatic β-cell function and a loss of sensitivity to insulin are often significant factors in the development of T2DM [144]. Whether patients deal with T1 or T2DM has little consequence on the host of complications that they are likely to encounter as a result of this cellular dysfunction, including cardiomyopathy, hypertension, cataracts, and more [145,146]. It has been discussed whether the significant variability in the severity of these clinical outcomes of diabetes could be due to Ca^2+^ dysregulation. Decades ago, Levy and colleagues [147] stated that in the majority of tissues observed (i.e., heart, erythrocytes, platelets, skeletal muscle, kidney, hepatocytes, aorta, adipocytes, liver and osteoblasts), whether involving human diabetes patients or animal models for the disease, exhibited increased intracellular Ca^2+^ concentrations often accompanied by decreased Ca^2+^-ATPase activity. These findings highlight that deregulated Ca^2+^ homeostasis is a fundamental disorder in the diabetic state.

Much of the literature investigating a potential role for the ion in diabetes focuses on dietary Ca^2+^, taken either alone or in consideration with other important components of a healthy diet, such as magnesium (Mg^2+^) or vitamin D [148,149,150]. Increased dietary Mg^2+^ intake, for example, was found to correlate with a decreased risk of T2DM in subjects, while increased consumption of Ca^2+^ appeared not to show any association with risk [148]. It is well-outlined, for example, that vitamin D functions in the maintenance of phosphorus and Ca^2+^ homeostasis, thus promoting the healthy mineralization of bone. Recently, however, vitamin D and Ca^2+^ together have begun to be considered as factors affecting an individual’s risk of diabetes [149]. Namely, the existence of a link between vitamin D deficiency and downstream irregularities in glucose-induced release of insulin, a Ca^2+^-dependent process [151], appear to be connected by the ion itself. Similarly, a project investigating the regulation of blood sugar control in T2DM patients demonstrated that the addition of vitamin D, with or without supplemental Ca^2+^, improved glycemic status [150]. Vitamin D deficiency is causing β-cell death and, thus, is contributing to the development of β-cell dysfunction and β-cell death as onset and progression of diabetes. This vitamin not only maintains normal resting levels of Ca^2+^ and reactive oxygen species (ROS) [152]—both factors which are deregulated in diabetes [147,153,154]—but also prevents DNA hypermethylation of gene promoter regions regulating the expression of diabetes related genes [155].

Such research implies, therefore, that if Ca^2+^ is involved in the pathophysiology of T2DM, then it is not a matter of maintaining a certain level of dietary intake of the mineral, but rather has to do with an ingrained physiological signaling role of the ion. Indeed, it has been determined that normal Ca^2+^ homeostasis is impaired in patients with T2DM. Irregularities have been observed in a range of cell types, including platelets, cardiomyocytes, and pancreatic β-cells, among many others [156,157,158]. The widespread observance of altered steady-state Ca^2+^ levels indicates that this serves as a consistent factor in diabetic conditions.

Ca^2+^’s proper functioning across multiple signaling pathways is vital in all cells but is also important in some circumstances due to a tissue-specific role. Also important is the tight interconnectivity of Ca^2+^ signals across different cellular pathways and mechanisms, wherein a disturbance in one could affect a host of others. A dysregulation in such a signaling web oftentimes makes diagnoses of the original problem difficult, if not impossible. Undeniably, multiple changes to normal Ca^2+^ signals and regulation have been described in diabetes patients and animal models, hitting all of the main players in Ca^2+^ movement, such as SERCA and NCX, across tissues [147,156]. Thus far, the most agreed-upon abnormality in Ca^2+^ homeostasis in diabetes appears to be an increase in steady-state cytosolic Ca^2+^ levels across different cell types [147].

More specifically, in β-cells, the Ca^2+^ dysregulation evident in diabetes is so far better characterized in animal and cell-level models of the disease. Changes to normal Ca^2+^ homeostasis include increased voltage-dependent Ca^2+^ channel activity in diabetic rodents as well as decreased SERCA activity [157,159]. Considering the bigger picture, it is known that glucose homeostasis is achieved predominantly through many pathways that are regulated by Ca^2+^, including glycolysis, gluconeogenesis, and others [160]. These vital processes are thus derailed if some form of Ca^2+^ dysregulation event occurs, which could be the end result of changes to normal Ca^2+^ channel or pump activity. For example, as mentioned, an increased intracellular Ca^2+^ concentration is evident in diabetes which results in the inhibition of glycogen synthase, ultimately causing, through a disrupted series of activations and phosphorylation events, glucose resistance [147].

**Figure 2:**
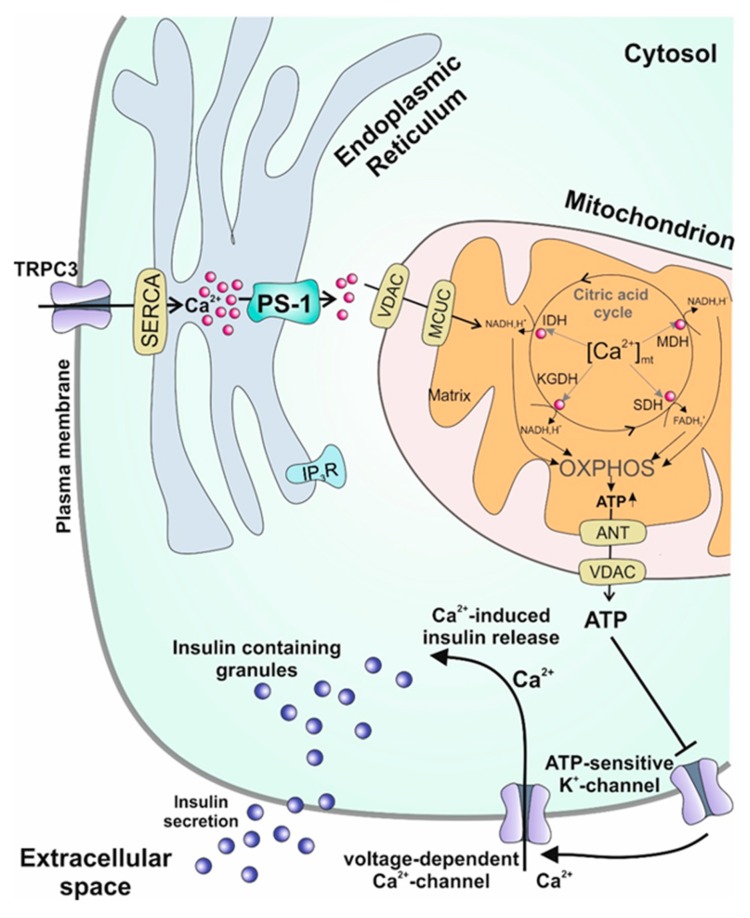
Graphical summary of the consequences of the β-cell-specific presenilin-1-mediated ER Ca^2+^ leak. Ca^2+^ leaking out of the ER is directly sequestered to mitochondria, leading to increased basal matrix Ca^2+^ levels, where it pre-stimulates the Ca^2+^-dependent dehydrogenases of the citric acid cycle, augmenting resting organelle ATP levels. ATP-sensitive K^+^-channels are inhibited leading to cellular depolarization. This electrochemical shift triggers Ca^2+^ uptake via L-type Ca^2+^ channels. As a result, Ca^2+^-induced Ca^2+^ release is initiated, promoting insulin exocytosis into the extracellular space.

As the vigilant reader of the manuscript might have already realized, a new and for some maybe pugnacious finding concerning β-cell responsiveness and regulation of insulin secretion is presented in several sections of the manuscript—namely, the crucial role of a presenilin-1-mediated ER Ca^2+^ leak for proper β-cell function [28,107], data which are graphically summarized in Figure 2.

Our work demonstrates that this leak is regulating many steps in the cascade ultimately leading to insulin secretion. Reducing the ER-Ca^2+^ leak by either knocking down presenilin-1 or inhibiting its activity by blocking the PS1-regulatory kinase GSK3β causes several important effects in β-cells: i. reduction of mitochondrial respiration; ii. decreased mitochondrial ATP levels; iii. reduction of glucose-stimulated Ca^2+^ oscillation and delayed onset of these oscillations; iv. abrogation of the first-phase of insulin secretion. Several studies have already demonstrated that a loss of the first phase insulin response [95] which is resulting in delayed insulin secretion [161,162,163,164] is an irregularity occurring in the early stages of T2DM. The loss of this first phase is worsening post-prandial hyperglycemia yielding a progression of the pathologic condition and ultimately leading to clinically relevant hyperglycemia [165]. Therefore, this loss of first-phase insulin secretion is a predictive marker for the risk of developing T2DM [166,167], highlighting the importance of a regulated biphasic insulin response. The results of our study demonstrate that a functional ER-mitochondrial Ca^2+^ transfer mediated by presenilin-1 is pivotal for an adequate insulin response [28,107].

## 6. Presenilin-1, the Missing Link between Diabetes and Alzheimer’s Disease?: Excursus

In this section, we would like to draw the attention to a circumstantiated but sometimes underestimated correlation of two diseases where both presenilin-1 and Ca^2+^ are known contributors to disease development namely T2DM and Alzheimer’s disease (AD). Interestingly, there is a profound epidemiologic and experimental correlation between these two devastating diseases. Several studies show that T2DM may lead to cognitive impairment [168,169]. Until recently the amyloid theory which states that β-amyloid plaques cause apoptotic cell death of neuronal cells was the most supported theory as a causative trigger for AD development [170,171,172]. This theory finds support by several studies demonstrating the occurrence of increased Aβ42/Aβ40 ratios in the early stages of AD development [173]. At this point the presenilin-1 comes into play: together with PEN-2, APH1 and nicastrin, presenilin-1 forms the y-secretase complex [174,175] responsible for the production of β-amyloids. Since β-amyloid plaque formation is considered a hallmark of AD, y-secretase function is implicated in the pathogenesis of AD [176]. New discoveries challenge this hypothesis [176,177,178] by demonstrating that mutations of presenilin-1—which is pivotal to Aβ production—do not increase the activity of the protein but rather decrease it. A phenomenon observed in vitro and in cells [179,180,181,182,183] and also enzymatic inhibition fails to reduce symptoms of AD patients [184]. Moreover, as β-amyloid plaques are also found in healthy individuals [185,186] and patients suffering from front temporal dementia (FTD), a disease with similar functional defects but a lack of plaque formation [187,188,189], the correlation between the occurrence of plaques with the emergence and severity of AD is only weak. All these studies indicate that the y-secretase/amyloid theory is not the only causative factor for AD development. At this point the Ca^2+^ comes into play: the more than 219 AD-associated mutations in the presenilin-1 gene [190] are the most common cause of the inherited form of AD, namely familial AD (FAD) that can onset already early in life. In contrast, the more common form of AD, from those a possible influence of presenilin mutations has not established so far, develop later in life. Notably, all so far tested presenilin-1 mutations also influence in some aspect intracellular Ca^2+^ handling [191,192,193]. Depending on the mutation(s) present both possible functions of presenilin-1 can be altered i.e., y-secretase activity and Ca^2+^ handling. Examples, therefore, are the following: ΔE9 affects y-secretase function, M146V disturbs ER Ca^2+^ handling, L166P affects both [194]. Accordingly, deranged Ca^2+^ homeostasis and β-amyloid toxicity due to mutations of presenilin-1 might act as independent causative factors for developing FAD. Importantly, the known crucial involvement of presenilin-1 in physiological amyloid degradation [195] and its essential function for β-cell responsiveness [28,107] might explain the high incidence of T2DM in AD patients. This possible link could connect the high incidence of the non-inherited version of AD and T2DM. Concluding this paragraph, changes in Ca^2+^ homeostasis due to alterations in presenilin-1 function could be the missing link for the causative factors of diabetes and AD development and could shed some light into the correlation of these two diseases. The exact regulation as well as additional mechanisms still need to be further elucidated but these data justify more intensive studies on this topic.

## 7. Discussion and Conclusions

Considering the implications of the intracellular changes discussed here, it is clear that further investigation into the Ca^2+^ signaling and other machinery of β-cells is required to truly gain insight into how they relate to the pathophysiology of diabetes. Researchers are constantly innovating new techniques to accomplish exactly this. For example, the concept of synthetic cells has been around for decades [196,197], but recent work has made significant strides. Groups have so far reported using vesicles seeded with integrated proteins for various purposes, and nanoparticles covered in cell-membrane materials, among other creative cell facsimiles [198,199] to mimic vital and cell-specific processes. The development of synthetic β-cells, in particular, could prove to be a promising technique for gaining an in-depth understanding of the dynamics of insulin secretion and other physiological processes. The concept, as is it has so far been developed, involves using synthetic materials in order to mimic glucose-dependent secretion of insulin, an application that could prove important for making strides in diabetes research. Chen and colleagues [200] have recently made headway with this notion, using the structure of ‘vesicles-inside-a-vesicle’ with additional features that allow for glucose metabolism and the ability of membranes to fuse. These artificial cells have so far been shown to differentiate between varied experimental glucose conditions. When glucose is high, the artificial β-cells exhibit high glucose uptake and increased oxidation, resulting in a decreased pH within the compartment. Low pH subsequently induces mechanical changes within the cells’ that result in a fusion of inner and outer vesicle membranes, thus simulating insulin release through exocytosis. While still a rudimentary system, such work could ultimately contribute to the emergence of cell therapy as a potential course of action in diabetes; countering the worst of the limitations of the current practice of islet donation.

While promising concepts are constantly emerging, it is vital to continue diabetes investigation with the tried and tested cellular and animal models available to all. As we have outlined here, recent work has helped identify specific mechanisms of intra- and inter-cellular communication that allow for the complicated insulin-secreting function of β-cells, particularly considering the importance of Ca^2+^ to such processes. Despite now knowing of the intricate roles that this ion plays in pancreatic cell-specific function, there is a significant knowledge gap between early questions posed on Ca^2+^’s importance to the development of diabetes and current understanding of the same query.

The research discussed here reveals a significant phenomenon in pancreatic β-cells that sheds further light on the mechanism by which these cells exhibit a response to increased glucose levels. Dysregulation of this mechanism could therefore potentially contribute to the pathophysiological development of T2DM or could be the missing link to other diseases.

## Abbreviations

AD	Alzheimer’s Disease
ATP	Adenosine triphosphate
CaMK	Ca^2+^/calmodulin dependent kinase
CICR	Calcium induced calcium release
DM	Diabetes mellitus
ER	Endoplasmic reticulum
GLUT	Glucose transporter
GSIS	Glucose stimulated insulin secretion
GSK3β	Glycogen synthase kinase 3 β
IP_3_	Inositol trisphosphate
IP_3_R	IP_3_ receptor
IR	Insulin receptor
IRS	Insulin receptor substrate
MCU	Mitochondrial calcium uniporter
MICU	Mitochondrial calcium uptake 1
NCX	Na^+^/Ca^2+^ exchanger
NCLX	Mitochondrial Na^+^/Ca^2+^ exchanger
NFAT	Nuclear factor of activated T-cells
PM	Plasma membrane
PMCA	Plamsa membrane Ca^2+^-ATPase
PTP	Permeability transition pore
RyR	Ryanodine receptor
SERCA	Sarco/endoplasmic reticulum Ca^2+^-ATPase
T1/2DM	Type 1 or type 2 diabetes mellitus
VDCC	Voltage dependent calcium channels
